# The Effectiveness of Debriefing on the Mental Health of Rescue Teams: A Systematic Review

**DOI:** 10.3390/ijerph22040590

**Published:** 2025-04-09

**Authors:** Francesca Ancarani, Pedro Garijo Añaños, Bain Gutiérrez, Juan Pérez-Nievas, Germán Vicente-Rodríguez, Fernando Gimeno Marco

**Affiliations:** 1 Catedra de Montaña, University of Zaragoza, Ayuntamiento de Huesca y Diputación Provincial de Huesca, 50009 Zaragoza, Spain; fancarani@unizar.es (F.A.); fergimen@unizar.es (F.G.M.); 2 EXER-GENUD (Growth, Exercise, Nutrition and Development) Research Group, Universidad de Zaragoza, 50009 Zaragoza, Spain; 3 Centro de Adiestramientos Específicos de Montaña (CAEM), Servicio de Montaña, Guardia Civil, Ministerio del Interior, 22889 Candanchu, Spain; 4 Grupos de Rescate Especial de Intervención en Montaña (GREIM), Servicio de Montaña, Guardia Civil, Ministerio del Interior, 22700 Jaca, Spain; 5 Urgencias y Emergencias Sanitarias 061 Aragón, SALUD Aragón, Gobierno de Aragón, 50009 Zaragoza, Spain; 6 Centro de Investigación Biomédica en Red de Fisiopatología de la Obesidad y Nutrición (CIBEROBN), Instituto de Salud Carlos III, 28040 Madrid, Spain; 7 Instituto Agroalimentario de Aragón (IA2), Universidad de Zaragoza-CITA, 50013 Zaragoza, Spain; 8 Department of Physiatry and Nursing, Faculty of Health and Sport Sciences, University of Zaragoza, 50009 Zaragoza, Spain

**Keywords:** psychological debriefing, rescue teams, post-traumatic stress disorder (PTSD), post-traumatic interventions, mental health

## Abstract

**Background**: Rescue teams and emergency services face high levels of mental health problems due to their frequent exposure to traumatic situations. Critical incident stress debriefing (CISD) is widely used as a psychological intervention for emergency responders and military personnel exposed to traumatic events. However, its effectiveness remains controversial, with systematic reviews yielding mixed results and some evidence of negative and harmful outcomes. This systematic review, conducted according to PRISMA guidelines, evaluates the evidence on the efficacy of CISD in mitigating psychological distress and preventing post-traumatic stress disorder (PTSD). **Methods**: A systematic search was conducted in PubMed and PsycINFO from inception to November 2024. Eligibility criteria included randomized controlled trials (RCTs) and cohort studies assessing the impact of CISD on PTSD, anxiety, depression, and psychological distress. Two independent reviewers screened studies, extracted data, and assessed the risk of bias using the PEDro scale. Data narrative synthesis was applicable. **Results**: A total of 6 out of 371 studies were included, comprising 4751 participants. The PEDro scale showed that one study was of high methodological quality, four were of acceptable quality, and two had deficiencies. The findings revealed mixed outcomes: while some studies reported a reduction in PTSD symptoms, others found no significant effect or even potential harm. Heterogeneity in intervention implementation, population characteristics, and study quality influenced the results. Risk of bias was moderate to high in several studies, with limitations in sample size and follow-up duration. No specific effects have been studied in mountain rescue teams. **Conclusions**: Current evidence does not unequivocally support the efficacy of CISD in preventing PTSD and psychological distress. Given methodological concerns and potential adverse effects, alternative debriefing methods, such as Battlemind debriefing, warrant further exploration. Future research should focus on well-powered RCTs with standardized intervention protocols to enhance reliability.

## 1. Introduction

Rescue teams are composed of professionals trained to respond to emergencies and relief situations. These teams include police groups (involved in security management in crisis situations and assisting in rescue and evacuation tasks), soldiers (combining their military skills with specialized life-saving techniques in emergency situations, providing assistance in search, rescue, and evacuation operations in high-risk or conflict environments), firefighters (responsible for extinguishing fires, rescuing people in dangerous situations, and handling hazardous materials), paramedics (providing emergency medical care and stabilizing patients in emergency situations, providing emergency medical care and stabilizing patients in dangerous situations, rescuing people in dangerous situations, and handling hazardous materials), paramedics (providing emergency medical care and stabilizing patients before transporting them to health facilities), and search and rescue personnel (specializing in locating and rescuing trapped or lost people, often in difficult terrain such as mountains, forests, or collapsed areas) [1,2].

These professionals face various incidents, each with its own challenges and risks. In the case of natural disasters, such as earthquakes, they must deal with building collapses, which trap people and cut off basic services. Floods require the rescue of people trapped in flooded homes and the evacuation of at-risk areas. On the other hand, hurricanes and storms can cause extensive damage to infrastructure, requiring the search for survivors in debris. Forest fires involve fire control, extinguishing, and the rescue of people and animals.

Major accidents are another frequent type of incident. Traffic accidents, for example, may involve multiple collisions and the rescue of victims trapped in vehicles, in addition to providing first aid at the scene. In industrial accidents, on the other hand, rescue teams must handle hazardous materials and perform rescues in high-hazard environments, such as factories and chemical plants. Likewise, air and rail accidents require survivors to search for, rescue, and manage scenes with multiple victims.

In addition, rescue teams respond to medical emergencies, such as heart attacks, where rapid response for patient stabilization and transport is crucial. Similarly, drug overdoses demand urgent treatment and transport to medical facilities, while serious injuries require immediate patient care and stabilization.

Due to the nature of their work, rescue professionals are constantly exposed to high-stress and traumatic situations. The frequency and severity of incidents, hazardous working conditions, and the emotional impact of rescuing or failing to rescue victims contribute to this exposure. In addition, long shift workloads and lack of adequate rest and recovery increase fatigue and stress [3,4].

The analysis of mental health support mechanisms, such as debriefing, for rescue teams is intrinsically linked to environmental factors and public health outcomes. Rescue teams often operate in high-stress environments characterized by natural disasters, public health emergencies, and other crises. The psychological toll of these situations can lead to significant mental health challenges, including anxiety and post-traumatic stress disorder (PTSD), which not only affect the responders but also influence their effectiveness in providing care to affected populations [5,6].

The aforementioned high exposure to traumatic and stressful events can have various consequences for these professionals. Apart from anxiety disorders, depression, and severe sleep problems, two of the most common complications are post-traumatic stress disorder (PTSD) and burnout [7].

PTSD is a mental disorder that can develop after a person has been exposed to a traumatic event [1]. However, experiencing a tragic event is not the only way to suffer traumatic stress. People who help in an emergency may suffer symptoms associated with PTSD, even though they did not experience the stressful event firsthand. Secondary traumatic stress is a very common psychological condition in people doing humanitarian work [8]. Secondary traumatic stress is defined as a psychological condition in which negative emotions and behaviors occur upon learning of a traumatic event experienced by another person. That is, it occurs when a person who frequently works with people who have been affected, usually in the humanitarian sector, is affected by the pain of others in a pathological way. This psychological phenomenon is also known as vicarious traumatization.

Since rescue teams are typically deployed after an event rather than during it, their arrival can sometimes trigger it. This delay may heighten fear and reinforce a sense of ongoing threat, making the experience even more distressing.

In rescue teams, PTSD symptoms are particularly common due to the nature of their work [9,10]. These symptoms may include reliving the traumatic event through flashbacks, nightmares, or intrusive thoughts about the event [11,12,13]. In addition, people may experience avoidance, i.e., they avoid places, people, or activities that remind them of the traumatic event. Hyperarousal is another symptom, which manifests with insomnia, irritability, difficulty concentrating, and an exaggerated startle response [13]. Negative changes in thinking and mood are also common, including persistent negative thoughts about oneself or the world, feelings of guilt or shame, and a loss of interest in previously enjoyable activities [11,12,13].

Although the prevalence of PTSD among police officers acting during the terrorist attack in Madrid was only about 1.3% [14], results from The House of Commons indicate that the prevalence on rescue teams internationally varies between 10% and 35% [2], with some studies showing it to be significantly higher compared to the general population [15,16]. For example, some research has revealed that 45% of firefighters have experienced four or more potentially traumatic events [17] and about 32% of them have clinically significant PTSD symptoms [18]. In 2022, approximately 72,965 cases of PTSD were reported in Spain [19].

Burnout is another prevalent mental health problem among rescue workers [20]. It is characterized by extreme emotional and physical fatigue, depersonalization, and diminished personal accomplishment [21,22]. Professionals suffering from burnout may feel physically and emotionally exhausted and may develop a cynical or distant attitude toward their work and the people they serve. In addition, they may experience a reduction in their work performance, with decreased efficiency and productivity and an increased propensity to make mistakes [21].

These mental health problems have a significant impact on the individual well-being of rescue professionals and their professional performance [23]. PTSD can lead to long-term health problems, difficulties in personal relationships, and an inability to perform everyday tasks [24]. Burnout can result in a decrease in the quality of service provided, increased absenteeism, and increased staff turnover. Taken together, these problems affect the health and well-being of professionals and can have negative consequences for rescue organizations and the community at large, which depends on these essential services.

For this reason, it seems crucial that rescue teams receive adequate psychological support to help them process traumatic experiences, reduce the effects of stress, and maintain their mental health and general well-being. One of the most commonly employed therapeutic approaches in this regard is cognitive behavioral therapy (CBT), which has proven to be effective in the treatment of PTSD and other anxiety disorders [25,26]. CBT focuses on changing negative thought patterns and developing effective coping strategies. In addition, resilience programs and stress management training have also been implemented to strengthen the ability of rescue professionals to manage stress and reduce the risk of burnout [27].

Despite this variety of alternatives, techniques such as debriefing have been created to address these issues in rescue team members.

### 1.1. Debriefing as a Psychological Intervention in Rescue Teams

Debriefing is a structured group technique based on the critical incident stress debriefing (CISD) model [28,29], which was initially created as a short-term, group-format, preventive mental health intervention for law enforcement and emergency services personnel who had experienced traumatic situations. Since its origin, the debriefing model has been adapted and modified to apply to various clinical groups and crisis contexts, leading to changes in its structure and procedures [30]. It is applied to help process events and lived experiences after a traumatic event [30]. In mountain rescue teams, these experiences often have a high emotional impact due to the serious mishaps that occur during rescue operations and could have special connotations [31].

We acknowledge that it is a controversial topic since CISD has faced considerable scrutiny and is reported to have negative effects, contributing to its elimination from some jurisdictions. Some evidence indicates that while intended to alleviate stress reactions, CISD may be ineffectual and, in some instances, harmful to participants [32,33]. Research findings have highlighted that CISD can potentially exacerbate symptoms of PTSD rather than mitigate them, prompting authorities to reconsider its use in mental health interventions following traumatic events [32,34,35]. However, it may be due to the application method more than the method itself [36], which has been used and investigated, and more research is required according to different contexts.

The debriefing event for analysis and recovery group intervention is designed to reduce emotional impact and burnout [37] and prevent future mental health problems, such as PTSD. The main goals of debriefing are to provide a safe space for emotional expression, normalize reactions to trauma, and foster mutual support among participants.

The debriefing process consists of several phases, each with a specific purpose [29]. The session begins with an introduction, where the purpose of the meeting is explained, and the rules of confidentiality and respect are established. Next, the facts of the event are reviewed, allowing participants to describe the incident from their perspective and focus on objective details. Subsequently, participants’ reactions and thoughts are discussed, allowing them to express their emotions about the event. Next, symptoms experienced since the incident, both physical and emotional, are addressed. In the teaching phase, information about common reactions to trauma is provided, and coping strategies are offered. Finally, in the re-entry phase, the session concludes with a summary and information about additional resources, and follow-up is provided [29].

There are several types of debriefing tailored to the needs of the group and the nature of the traumatic event. One of the best-known is critical incident stress debriefing (CISD), developed by Jeffrey T. Mitchell [28,29] (a psychologist and former firefighter worried about the absence of structured psychological support systems for first responders), which focuses on emergency teams and other high-risk groups. The key features of this model are the structured process—CISD follows a seven-phase protocol, including fact-sharing, emotional reactions, symptom discussion, teaching of coping strategies, and re-entry into normal activities [29]. It uses a group-based approach and is conducted in small peer groups, typically facilitated by a trained professional. It is preventative and educational—it is not therapy but rather a structured discussion meant to help individuals process emotions and normalize responses. Last, it is used within the CISM framework—CISD is part of a broader critical incident stress management (CISM) system, which includes pre-incident education, peer support, and professional counseling if needed.

Jeffrey T. Mitchell’s CISD model [29] is one of the most widely applied theories. Developed for emergency teams and other groups that regularly face traumatic situations, CISD’s main objectives are to mitigate the emotional impact, help participants process and manage immediate emotional reactions, normalize reactions by providing information that helps them understand that their responses are normal given the circumstances, and foster group support by creating an environment where participants can share experiences and offer mutual support.

### 1.2. Other Debriefing Models and Interventions

Social support theory stresses the importance of support networks in the management of stress and trauma. According to this theory, social support can provide emotional resources, facilitate emotional expression safely and constructively, and improve people’s coping capacity [38,39]. In the context of debriefing, this theory highlights how a supportive environment, whether among colleagues, friends, or family, can offer comfort and reduce feelings of isolation, thus contributing to the well-being and recovery of people affected by trauma. Another type is psychological debriefing, which is more widespread and is used in a variety of clinical settings, adapting to different groups affected by trauma [40]. There is also informational debriefing, which is less structured and can occur spontaneously among colleagues or friends after a traumatic event [41]. Thus, intuitively, debriefing could act as a preventive measure against possible future mental disorders, such as post-traumatic stress disorder, and could contribute to individual and group health, thus improving the performance of professionals and rescue teams [42].

Battlemind debriefing is another intervention tailored for military personnel returning from combat. It serves as both a therapeutic debriefing and a psycho-educational program, aiding soldiers in processing traumatic experiences, and thereby facilitating their transition back to civilian life. The intervention is grounded in helping participants comprehend their responses to trauma and the potentially maladaptive behaviors acquired during deployment, such as hypervigilance [43,44]. Research indicates that the Battlemind approach effectively reduces PTSD symptoms and depression, particularly among service members with extensive combat experience. Notably, participants who underwent Battlemind training exhibited fewer mental health issues compared to those receiving standard stress education after four months [45,46]. However, while some studies suggest its effectiveness, particularly for those with high levels of combat exposure, critics highlight that further research is needed to understand its impact comprehensively [47,48]. Overall, Battlemind debriefing represents a valuable component in the spectrum of mental health support for military personnel, aimed at fostering resilience and aiding successful reintegration into civilian life.

The 512 psychological intervention model (512 PIM) was developed to assist military rescuers following the Wenchuan Earthquake in China. This model integrates aspects of CISD with an emphasis on unit cohesion and social support, both of which are supposed to mitigate stress-related conditions such as PTSD [49]. The 512 PIM model is characterized by a systematic approach involving interventions that began shortly after the traumatic events, aimed at reducing symptoms of PTSD, anxiety, and depression among military personnel [50,51]. Research suggests that those participating in this specific intervention showed significant improvements in their mental health outcomes compared to those who received traditional debriefing or no intervention at all [49,51]. A randomized controlled trial reported by Wu et al. involved 2368 military rescue personnel and indicated that the 512 PIM was effective in reducing PTSD symptoms over a follow-up period [49].

### 1.3. Debriefing Efficacy

Numerous studies have evaluated the efficacy of debriefing in mental health, with most studies focusing on people who have experienced a traumatic event. The findings have been mixed, and the studies appear to have serious methodological shortcomings [52,53]. However, rescue teams frequently exposed to traumatic events have special characteristics that differ from the general population, including extensive training and specialization in emergency situations, so they may respond differently to debriefing strategies previously detailed. However, the effectiveness of these approaches may vary depending on the specific needs and resilience of the person. As far as we know, these aspects have not been analyzed in detail in rescue teams. Some studies suggest that debriefing can help reduce post-traumatic stress symptoms and improve emotional well-being in this population [42]. For example, it has been found to provide a safe space for professionals to express their emotions and process their experiences, which may reduce the immediate emotional impact and prevent long-term mental health problems [42]. However, in that study, conclusions are drawn, again from civilian victims and not rescue groups, since, as the authors conclude, only 1 of 15 studies included in their review was conducted on emergency personnel [42]. Therefore, knowing the effect of debriefing on emergency personnel or rescue groups may help propose psychological support programs to prevent or treat PTSD or burnout in this specific population.

On the other hand, other research has questioned these benefits, indicating that debriefing may not be as effective as initially thought. Some studies even suggest that it may have negative effects, such as retraumatizing participants by reliving the traumatic event [54]. Additionally, systematic reviews and meta-analyses have pointed out the low quality of many debriefing studies, highlighting issues such as inadequate control groups, small sample sizes, and flawed study designs [55]. These methodological limitations [55] complicate drawing definitive conclusions about the efficacy of debriefing in the general population [52,54], and given the special characteristics of emergency and rescue professionals, warrant individualized study. We hypothesize that there are high controversies; therefore, conducting a systematic, rigorous, and updated review of the existing literature may shed some light to clarify the effectiveness of debriefing in this population, paying special attention to psychological problems that are highly prevalent among these professionals [7] and that have not been previously analyzed about the effectiveness of debriefing. A comprehensive evaluation will identify the strengths and weaknesses of this intervention, as well as the conditions under which it may be most effective. At the same time, it is essential to consider other interventions and strategies that have proven effective in the psychological support of rescue teams, such as those mentioned above.

Therefore, despite accumulating evidence of negative and some harmful outcomes of CISD to date, to further clarify its evidence base, particularly for rescue teams, the present research focuses on debriefing as an intervention aimed at mitigating mental health problems in rescue teams. The central question guiding this review is: Is debriefing effective for treating post-traumatic stress disorder or burnout in rescue teams compared to other interventions? In this regard, the main objective of this review is to evaluate the effectiveness of debriefing in preventing and treating these specific problems through a systematic review of the existing scientific literature.

## 2. Materials and Methods

This work is developed within the Debriefing and Psychological Support to Rescue Teams working group of the Mountain Chair of the University of Zaragoza. The systematic review will follow the PRISMA (Preferred Reporting Items for Systematic Reviews and Meta-Analyses) guidelines [56] and the Cochrane methodology [57], using the PICOS strategy for developing the research question [58]. Additionally, the review has been registered at PROSPERO (Prospero num. CRD42024618564).

The PRISMA methodology establishes guidelines for the complete elaboration of systematic reviews and meta-analyses. Its objective is to optimize the quality and transparency of the information presentation, ensuring that all the essential elements for assessing the validity and applicability of the results are included. PRISMA facilitates a clear and detailed structure that guides the process of formulating the research question to presenting the findings, thus promoting reproducibility and comparability between studies [56].

On the other hand, the Cochrane methodology focuses on conducting high-quality systematic reviews in healthcare. The Cochrane collaboration provides rigorous standards for designing, conducting, and interpreting evidence-based systematic reviews and meta-analyses. Its approach is based on thorough and critical identification of the relevant literature, meticulous assessment of the risk of bias in the included studies, and statistical synthesis of the results to provide clear and robust conclusions about the effectiveness of the evaluated interventions [57].

Both methodologies, PRISMA and Cochrane, complement each other to ensure that systematic reviews are conducted comprehensively and methodologically soundly, thus ensuring that the results are reliable and useful for clinical and health policy decision-making.

Finally, the PICOS strategy [(P: population or problem of interest; I: intervention; C: comparison; O: outcome; S: study)], is a tool used in research to formulate specific clinical questions and structure the search for relevant evidence.

### 2.1. Search Strategy and Databases

The search strategy, summarized in Table 1, was designed to identify relevant studies in the PubMed and PsycINFO databases. It used a combination of MeSH terms and keywords related to “debriefing”, “psychological support”, “rescue teams”, or similar terms, as well as specific mental health terms such as “PTSD” and “burnout”.

### 2.2. Inclusion Criteria

For articles to be included, they had to meet the following inclusion criteria based on the PICOS method:

- Population (P): members or participants in organized rescue groups;
- Intervention (I): debriefing;
- Comparator (C): control comparisons, placebo, or other conservative non-pharmacological interventions;
- Outcome variables (O): post-traumatic stress disorder and burnout;
- Type of study (S): clinical trials;
- Articles published in any year.

Quantitative studies investigating the effect of debriefing on rescue teams were included. The articles had to be published in English or Spanish and accessible through indexed scientific journals. When reported in studies whose main variables were PTSD or burnout, results of other variables associated with stress or psychological factors were also included.

### 2.3. Exclusion Criteria

We excluded studies that did not explicitly focus on debriefing in rescue teams, along with those that addressed disorders other than post-traumatic stress disorder (PTSD) and burnout. Qualitative and observational studies were also excluded, in addition to studies published in languages other than English or Spanish and in formats other than peer-reviewed scientific journals.

### 2.4. Selection Process

First, a review protocol was established to create a structured plan for the entire process to avoid potential biases and ensure the review’s transparency. In this step, the necessity of the study was clarified, and the review question was formulated. The search strategy was crafted, detailing the eligibility criteria, the data extraction and synthesis method, and the planning for disseminating the results.

Once the search had been carried out according to the previously defined strategy, duplicate articles were eliminated using the Mendeley bibliographic manager. The titles and abstracts of all the records obtained were read. A checklist was created in Microsoft Excel to evaluate whether each article met the inclusion and exclusion criteria and was relevant to our purpose.

Subsequently, the full articles preselected during the abstract review phase were read and analyzed. Using the checklist previously created, the inclusion and exclusion criteria were reapplied, thus selecting the articles to be included in the systematic review.

In summary, the process included the following steps:

*Initial search*: An exhaustive search was conducted in the databases above using the defined terms and strategies.

*Elimination of duplicates*: The Mendeley bibliographic manager was used to manage and eliminate duplicates efficiently.

*Title/abstract review*: The titles and abstracts of retrieved articles were examined to identify potentially relevant works.

*Full-text reading*: The articles selected in the previous stage were read completely to determine their final inclusion in the review.

*Article selection*: Studies that fulfilled the inclusion criteria were chosen for the systematic review.

*Data extraction*: Relevant data, including the characteristics of the studied population, the type of intervention and comparison, main results, and pertinent conclusions, were extracted from each selected study.

### 2.5. Methodological Quality of the Studies

The methodological quality of the studies was assessed using the PEDro scale, developed by Verhagen and colleagues through a Delphi study [59]. This scale comprises 11 criteria for evaluating the quality of clinical trials in systematic reviews. The 11 criteria are as follows:

1. The selection criteria were specified.
2. Participants were randomly assigned to groups.
3. The assignment was concealed.
4. The groups were similar at baseline.
5. All subjects were blinded.
6. All therapists were blinded.
7. All evaluators were blinded.
8. Measures of at least one key outcome were obtained from over 85% of the subjects initially assigned to the groups.
9. Results were provided for all subjects who received treatment or were assigned to the control group. When this was impossible, data for at least one key outcome were analyzed on an “intention-to-treat” basis.
10. Statistical comparisons between groups were reported for at least one key outcome.
11. The study provides measures of point and variability for at least one key outcome.

Despite being composed of 11 items, the total score is out of 10 because the first criterion is not included in the overall total. The PEDro scale indicates that a higher score reflects better methodological quality. Therefore, a score of 7 or higher is considered to indicate “high” quality, a score between 5 and 6 is deemed “acceptable”, and a score of 4 or lower is classified as “poor”.

## 3. Results

### 3.1. Results of the Review Process

**Figure 1:**
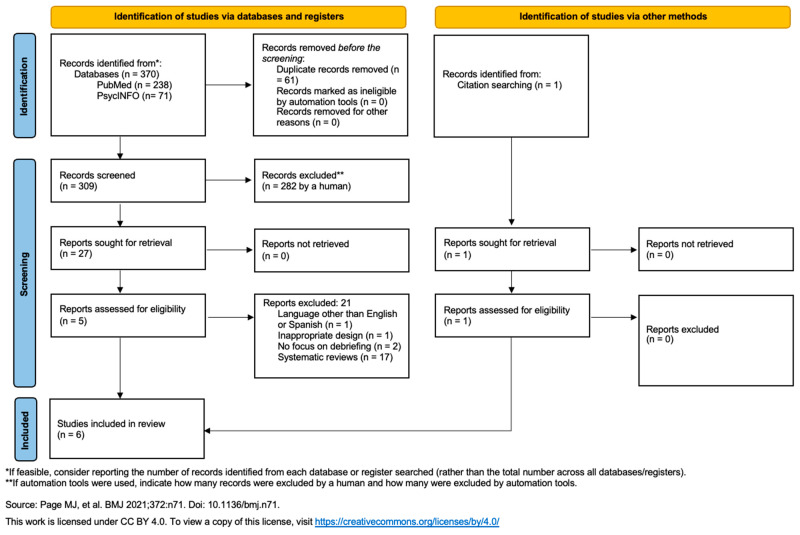
Flow chart according to PRISMA 2020 [60].

The initial literature search identified 370 studies, to which we added 1 study obtained through cross-referencing. Twenty-seven full articles were reviewed in detail, of which six met the inclusion criteria and were incorporated into the systematic review. Figure 1 shows the PRISMA flow diagram and why articles were excluded from the final selection of studies.

### 3.2. Results of the Methodological Quality Evaluation

Regarding assessing the methodological quality of the included articles using the PEDro scale, it was noted that the scores ranged from 4 to 7 points. Of the six studies evaluated, one was rated as high quality, three were classified as acceptable, and two demonstrated poor methodological quality (Table 2).

### 3.3. Main Results

The selected studies investigated the effects of debriefing, specifically through the structured CISD process in emergency teams. However, the study variables and populations are heterogeneous. In particular, three studies involved soldiers as participants, one involved Chinese military rescuers, one involved emergency personnel, and the last involved paramedics and emergency medical technicians (EMTs). These investigations were conducted across several countries, including the United States, Australia, England, China, and Canada. All studies examined the impact of debriefing on PTSD and, in some cases, also considered other variables such as psychological stress, quality of life, and alcohol consumption. No specific studies were found evaluating the effectiveness of debriefing in mountain rescue teams.

Adler et al. (2008) [61] conducted a cluster randomized trial with 952 peacekeepers, comparing critical incident stress debriefing (CISD) with a stress management class (SMC) and a survey-only (SO) condition. The results indicated that CISD did not accelerate recovery more than the other two conditions. However, among soldiers with greater exposure to mission stressors, CISD showed a slight reduction in reports of PTSD and aggression compared to SMC, as well as an increase in perceived organizational support compared to SO, although an increase in alcohol-related problems was also observed relative to both groups.

Tuckey and Scott (2014) [62] conducted a randomized clinical trial with 67 firefighters who had experienced a potentially traumatic event. Although CISD showed benefits in terms of lower alcohol consumption and improved quality of life compared with stress management education, no significant evidence was found that CISD was more effective in preventing PTSD or reducing psychological distress.

In the British Columbia Ambulance Service, a randomized controlled trial involving paramedics and medical technicians (EMTs) assessed three critical incident stress intervention strategies [64]. Over 26 months, 50 critical incident stress-related calls were documented, but only 18 participants enrolled, of whom 6 did not complete the forms. Although several outcomes were evaluated over a six-month timeframe, there was no consistent correlation between incident severity and stress scores, no clear pattern of stress reduction over time, and no participants who received formal debriefing. Due to low participation, the study could not adequately compare the three intervention levels, which was a key objective. However, the study suggests that while CISD is necessary, in the context of ambulance services, it likely does not require significant additional resource allocation.

Adler et al. (2009) [43] also compared various early interventions involving 2297 U.S. soldiers following their deployment in Iraq. In this context, they are not specifically rescue personnel, although their missions typically encompass these responsibilities, and in any case, they are exposed to high-stress situations. The study found that soldiers with significant combat exposure who underwent the Battlemind debriefing reported fewer symptoms of PTSD, depression, and sleep disturbances compared to those who received only stress education. Participants in smaller Battlemind groups also exhibited better outcomes for PTSD and sleep, while larger groups demonstrated fewer symptoms of depression and lower levels of stigma.

Deahl et al. (2000) [63] examined the psychiatric morbidity of 106 British soldiers returning from UN peacekeeping missions in the former Republic of Yugoslavia. All 106 soldiers received an operational stress (OS) training package before deployment. Immediately after their return from Bosnia, they participated in a formal psychological debriefing session following the Mitchell and Dyregrov method. Scores on the CAGE questionnaire for detecting drinking behaviors decreased significantly in the group that attended the briefing at the end of the follow-up period.

Finally, Wu et al. (2012) [49] compared the efficacy of a new psychological intervention model (“512 PIM”) with traditional debriefing in 2368 Chinese military rescuers. The results indicated that “512 PIM” was more effective in reducing symptoms of PTSD, anxiety, and depression than debriefing and the control group, demonstrating significant improvements in symptoms of re-experiencing, avoidance, and hyperarousal.

Taken together, these studies present mixed results on the effectiveness of CISD and other interventions, with some benefits noted in reducing PTSD symptoms and related issues but no clear consensus on their superiority compared to alternative strategies or the absence of intervention. Table 3 presents each study’s main characteristics and a summary of their main results.

## 4. Discussion

The findings from the reviewed studies offer new insights into the potential effectiveness of psychosocial interventions, including critical incident stress debriefing (CISD) and other techniques, in alleviating symptoms of post-traumatic stress disorder (PTSD), psychological distress, and other issues related to trauma.

Although some interventions, such as CISD, show positive effects in specific areas, like organizational support and quality of life, their effectiveness in preventing or alleviating PTSD symptoms or psychological distress is limited and varies by context and population. The results underscore the need for more personalized approaches, such as Battlemind training and 512 PIM, that are suited to the unique needs of individuals with varying levels of trauma exposure.

It is essential to differentiate between the various operational roles and tasks performed in the field, as they have distinct psychological and emotional impacts. Individuals engaged in armed protective duties, which necessitate the use of force to safeguard themselves and others, are more likely to exhibit heightened hostility and maladaptive anger responses [65]. Conversely, personnel responsible for providing medical assistance and first aid tend to experience a decline in quality of life (QoL) due to the emotional burden of caregiving, which is well documented for many health conditions [66]. These distinctions have significant implications for designing and implementing crisis intervention strategies.

Given these differences, the present review focuses on rescue teams, as their psychological responses and support needs differ fundamentally from those engaged in direct combat or military operations. While some included studies involve military personnel, they are limited to contexts where soldiers were deployed in rescue or humanitarian missions rather than active warfare. This ensures that the findings remain relevant to emergency responders, whose primary role involves providing aid rather than engaging in combat, thus aligning with the scope and objectives of this review.

CISD, as an intervention aimed at addressing the emotional and psychological effects following a traumatic event, may have been more applicable to those who experienced a higher level of stress. The intervention provided them with a structured environment to process the trauma, which seems to have helped in reducing symptoms of post-traumatic stress and aggression [61].

Furthermore, the CISD indicated that participants perceived more excellent organizational support than the SO condition. This could stem from the intervention’s group structure, which promotes teamwork and a sense of community, potentially enabling these soldiers to feel more connected and supported by their peers and the organization. This reinforces the findings of previous meta-analyses that identify social support post-incident as a critical factor in reducing the risk of developing PTSD [66]. Nevertheless, an increase in alcohol-related problems was noted in the group with greater organizational support, contrasting with the results of Tuckey and Scott’s (2014) [62] study, which demonstrated improved quality of life and reduced alcohol consumption among participants in the CISD group. A decrease in drinking behavior was also observed in the study by Deahl et al. (2000) [63], which examined the psychiatric morbidity of 106 British soldiers returning from UN peacekeeping missions in the former Republic of Yugoslavia. Scores on the CAGE questionnaire used to detect drinking behaviors decreased significantly in the group receiving psychological debriefing at the end of the follow-up period. Variations in the characteristics of the populations studied, their contexts, differing levels of stress exposure, and organizational culture may account for these discrepancies.

Tuckey and Scott’s (2014) [62] clinical trial was conducted with firefighters who had experienced a traumatic event and also used CISD as an intervention. Although the results showed improved quality of life and decreased alcohol consumption among participants in the CISD group, no strong evidence was found to indicate that this intervention or the others implemented were effective in preventing PTSD or reducing psychological distress. This study underscores the complexity of evaluating the impact of psychosocial interventions. It suggests that the positive effects of CISD may be limited and specific to certain outcomes rather than encompassing all aspects of mental health. While CISD appears to have improved some elements of general well-being and reduced risk behaviors such as alcohol use, it was not robust enough to have a profound impact on preventing PTSD or alleviating psychological distress. This implies that, although it may be useful as part of a broader emotional support approach, it may not be suitable as the sole intervention for severe psychological trauma.

On the other hand, studies such as that by Adler et al. (2009) [43] on Battlemind debriefing and Battlemind training in U.S. soldiers provide a more encouraging perspective. Results indicated that those with high levels of combat exposure who received Battlemind debriefing reported fewer post-traumatic stress symptoms, depressive symptoms, and sleep problems than those who received only stress education. The same effects were observed in participants of small-group Battlemind training with high levels of combat exposure. The results demonstrate that brief early intervention can be effective for at-risk occupational groups, emphasizing the importance of interventions that promote group cohesion and address participants’ specific experiences, such as combat exposure in this instance.

Finally, the study by Wu et al. (2012) [49], which evaluated a new psychological intervention (512 PIM) in Chinese military rescuers, found that this intervention was more effective than traditional debriefing in reducing symptoms of PTSD, anxiety, and depression.

The main distinction between the “512 PIM” and the debriefing lies in including a section dedicated to cohesion training. The “512 PIM” was initially designed for use in the Wenchuan seismic field, considering the practical principles and realities of the Chinese military organization. Due to several key factors, the “512 PIM” intervention likely proved more beneficial than traditional debriefing. First, the “512 PIM” includes a cohesion training section that fosters social support, mutual trust, and a sense of belonging among participants. This group cohesion may have played a significant role in reducing symptoms of PTSD, anxiety, and depression, as social support is a protective factor in trauma situations [38,39].

Moreover, the “512 PIM” was specifically designed by considering the Chinese military organization’s unique characteristics and the rescuers’ experiences, making it more suited to these groups’ needs and realities than traditional debriefing. This indicates that new approaches, such as the 512 PIM, could provide additional advantages over conventional interventions like CISD. Being tailored for populations facing intense trauma, such as rescue workers and military personnel, it may offer better adaptation to stressful situations and mitigate long-term psychological effects. Furthermore, since this model has demonstrated effectiveness in reducing symptoms of post-traumatic stress disorder (PTSD), depression, and anxiety, its implementation could lead to a significant decrease in the prevalence of these disorders among trauma-exposed workers, thereby playing a crucial role in their prevention. Although initially developed for military contexts, the 512 PIM holds the potential to be utilized in other sectors that encounter high levels of stress, such as emergency services, healthcare workers, and police forces.

In the future, if widely adopted, the 512 PIM could become a standard for post-traumatic stress management in high-risk teams, improving their mental well-being and crisis response capability.

Although we found little evidence to support the effectiveness of post-deployment or post-incident interventions, none of the studies reviewed reported adverse effects related to such interventions, except for the increased alcohol consumption noted by Adler et al. (2008) [61]. It is also undetermined whether this was a direct consequence of the intervention or an improved social climate.

This contrasts with earlier research suggesting that psychological debriefing, in some instances, is not only comparable to but also potentially less effective than educational or control interventions in preventing or reducing disorders such as PTSD, depression, anxiety, or general psychological morbidity. Some studies indicate that single-session debriefing may increase the risk of developing PTSD and depression, raising questions about its routine use in unselected trauma victims [54,67].

Consider the greater homogeneity and specialization of the participant groups in the studies selected for this review, which included soldiers, military rescuers, emergency personnel, paramedics, and emergency medical technicians (EMTs), all of whom were exposed to very similar traumatic situations or events, such as deployment in a common environment. However, the previous debriefing reviews mentioned above [54,67] included the general untrained population who had experienced trauma of varying natures.

The analysis presented in this review has several notable strengths. Firstly, it covers various interventions and population groups, providing a holistic view of the effectiveness of strategies such as debriefing and other measures designed to mitigate the impact of traumatic events. A more comprehensive understanding of how these interventions function in different high-pressure contexts is achieved by including military personnel, emergency responders, and healthcare workers. This breadth of analysis is essential, as not all populations respond in the same way to trauma, and understanding these differences can aid in tailoring interventions to the specific needs of each group.

Additionally, the review is not confined to a single intervention but compares various approaches, including critical incident stress debriefing (CISD), stress management (SMC), and innovative strategies such as the “512 PIM”. This comparison enhances the analysis by pinpointing the advantages and disadvantages of each intervention, offering a more nuanced and comprehensive perspective on their effectiveness.

Finally, the review is notable for examining positive outcomes and considering possible side effects. By pointing out, for example, the increase in alcohol consumption in some groups after debriefing, the review adds a critical layer of depth to the analysis, questioning the overall efficacy of the intervention and highlighting the importance of monitoring possible long-term risks.

However, some important limitations should be considered. We should keep in mind that perhaps some relevant MeSH terms were not identified, which could lead to a lower number of hits. Although several studies are included, the evidence on the effectiveness of the interventions is often limited or inconsistent. This complicates drawing definitive conclusions and sometimes diminishes the ability to generalize the results to various populations or contexts.

Furthermore, the heterogeneity of the methods used in the reviewed studies—across aspects such as design, duration of follow-up, type of intervention, and participant characteristics—complicates direct comparisons among them. This variability and the poor methodological quality of the studies, as indicated by the PEDro scale, may undermine the ability to draw solid conclusions regarding which intervention is more effective.

Another significant challenge is the difficulty in controlling for contextual variables. Factors such as the type of trauma experienced, differences in organizational culture, or specific characteristics of the groups studied are not always uniformly accounted for. This can significantly influence the results and restrict the ability to apply the findings to other contexts. Effective interventions in one setting may not be equally successful in another, highlighting the necessity to tailor interventions to the specific context.

A crucial factor is the lack of long-term follow-up in numerous studies. By failing to evaluate the effects of interventions beyond a few months, we miss the opportunity to understand whether the strategies employed have a lasting impact on PTSD, depression, anxiety, or overall well-being. This poses a significant issue, as the effects of trauma can unfold over many years, and the effectiveness of an intervention can only be fully evaluated if its long-term consequences are taken into consideration.

Finally, we should mention that although we are familiar with the PEDro scale, for simplicity, it is somewhat basic for the quality assessment of studies, and some others might prefer to explore this aspect in greater depth using other methodologies.

Future research on debriefing should concentrate on developing more homogeneous and controlled studies that address the methodological limitations and heterogeneity observed in this review. Trials with long-term follow-up are crucial for assessing the sustained effects of interventions, tailoring them to specific contexts, and considering factors such as organizational culture and type of trauma. Additionally, personalized and multimodal approaches, such as the 512 PIM, should be investigated to enhance the efficacy of debriefing.

### Public Health Perspective

From a public health perspective, the mental health of rescue teams is vital for ensuring a resilient healthcare response. Mentally fit rescue personnel are better equipped to deliver high-quality care and support to individuals experiencing crises [68]. Thus, integrating mental health support mechanisms into rescue teams’ operational protocols enhances their resilience and contributes to the overall effectiveness of public health responses in the face of environmental challenges.

## 5. Conclusions

In conclusion, despite accumulating evidence of negative and some harmful outcomes of CISD to date, this systematic review offers a comprehensive and nuanced perspective on the effectiveness of post-traumatic interventions, including debriefing and other strategies, in rescue teams subjected to high levels of stress. While some approaches, such as 512 PIM, have demonstrated promising results in specific contexts, the overall evidence regarding the efficacy of debriefing remains limited and inconsistent. Additionally, potential adverse effects, including increased alcohol consumption, were noted, highlighting the need for caution in the routine application of these interventions.

The heterogeneity in the studies reviewed, the lack of long-term follow-up, and the difficulty in controlling for contextual variables are significant limitations that hinder definitive conclusions from being drawn. In fact, there is a difference between someone having to protect themselves and others with weapons (which leads to more hostility and problematic anger) and someone having to provide first aid (which reduces QoL), which implies that both should be investigated in depth. Nevertheless, comparing various interventions and including diverse populations offers a valuable starting point for future research. We emphasize the need for more rigorous studies, employing context-specific approaches and longer follow-up periods, to more accurately assess the sustained impact of these interventions on psychological health. We cannot determine any specific effect on mountain rescue groups, as their impact on these particular groups has not been analyzed to date.

## Figures and Tables

**Table 1:** Search terminologies for each database.

Database	Participants AND	Intervention AND	Outcomes AND	Study Design AND
**Medline**	emergency personnel.tw emergency worker*.tw rescue worker*.tw first responder*.tw paramedic*.tw ambulance personnel.tw ambulance drive*.tw emergency responder*.tw emergency medical technician*.tw emergency medical service*.tw solider*.tw army.tw navy.tw police*.tw firefighter*.tw relief worker*.tw military.tw emergency services personnel.tw peacekeeper*.tw fire-fighter*.tw guardia civil.tw mountain rescuer*.tw mountain emergency.tw ICAR medcom.tw exp emergency medical services/ exp emergency responders/ exp emergency medical technicians/ exp law enforcement/ exp rescue work/ exp military personnel/ exp relief work/	post deployment.tw critical incident.tw post incident.tw trauma risk management.tw debrief*.tw exp secondary prevention/ exp crisis intervention/ exp disasters/	mental health.tw mental illness.tw mental disorder*.tw psychiatric.tw anxiety.tw depress*.tw mood disorder*.tw post-traumatic stress disorder.tw PTSD.tw traumatic stress disorder*.tw post-traumatic stress.tw psychological.tw stress*.tw exp anxiety disorders/ exp depressive disorder/ adjustment disorders.sh affective symptoms.sh anxiety.sh depression.sh mental disorders.sh mental health.sh neurotic disorders.sh	RCT.tw randomized controlled trial.tw random allocation.tw random assignment.tw randomization.tw randomly.tw randomized.tw quasi-experiment*.tw quasiexperiment*.tw control group.tw control condition.tw exp randomized controlled trial/ exp controlled clinical trial/ exp clinical trial/ exp random allocation/
**PsycINFO**	emergency personnel.ab,ti emergency worker*.ab,ti rescue worker*.ab,ti first responder*.ab,ti paramedic*.ab,ti ambulance personnel.ab,ti ambulance drive*.ab,ti emergency responder*.ab,ti emergency medical technician*.ab,ti emergency medical service*.ab,ti soldier*.ab,ti army.ab,ti navy.ab,ti police*.ab,ti firefighter*.ab,ti military.ab,ti relief worker*.ab,ti emergency services personnel.ab,ti fire-fighter*.ab,ti peacekeeper*.ab,ti exp law enforcement personnel/ exp military personnel/ exp emergency services/ exp first responders/ exp allied health personnel/ exp rescue workers/ guardia civil.ab,ti mountain rescuer*.ab,ti mountain emergency.ab,ti ICAR medcom.ab,ti	debrief*ab,ti post deployment.ab,ti critical incident.ab,ti post incident.ab,ti trauma risk management.ab,ti exp “debriefing (psychological)”/ exp disasters/	mental health.ab,ti mental illness.ab,ti mental disorder*.ab,ti psychiatric.ab,ti anxiety.ab,ti depress*.ab,ti mood disorder*.ab,ti post-traumatic stress disorder.ab,ti PTSD.ab,ti traumatic stress disorder*.ab,ti post-traumatic stress.ab,ti psychological.ab,ti stress*.ab,ti exp affective disorders/ exp anxiety/ exp anxiety disorders/ exp major depression/ exp “depression (emotion)”/ exp mental disorders/ exp neurosis/ exp PTSD/ mental health.sh. adjustment disorders.sh.	RCT.ab,ti randomized controlled trial.ab,ti random allocation.ab,ti random assignment.ab,ti randomization.ab,ti randomly.ab,ti randomized.ab,ti quasi-experiment*.ab,ti quasiexperiment*.ab,ti control group.ab,ti control condition.ab,ti exp treatment effectiveness evaluation/ exp experimental design/ exp mental health program evaluation/

* different terminantions

**Table 2:** Methodological quality according to the PEDro scale.

Study	Criteria											Total (Sum of 2 to 11)
	1	2	3	4	5	6	7	8	9	10	11	
Adler et al. (2008) [61]	0	1	0	1	0	0	0	0	1	1	1	5
Tuckey and Scott (2014) [62]	1	1	0	0	0	0	0	0	1	1	1	4
Deahl et al. (2000) [63]	1	1	0	1	0	0	0	0	1	1	1	5
Macnab et al. (2003) [64]	1	1	0	0	0	0	0	0	1	1	1	4
Adler et al. (2009) [43]	1	1	0	0	0	0	0	1	1	1	1	5
Wu et al. (2012) [49]	1	1	0	1	0	0	1	1	1	1	1	7

The 11 criteria are as follows: 1. The selection criteria were specified. 2. Subjects were randomly assigned to the groups. 3. Assignment was concealed. 4. Groups were similar at baseline. 5. All subjects were blinded. 6. All therapists were blinded. 7. All evaluators were blinded. 8. Measures of at least one of the key outcomes were obtained from more than 85% of the subjects initially assigned to the groups. 9. Results were presented for all subjects who received treatment or were assigned to the control group, or when this could not be done, data for at least one key outcome were analyzed on an “intention-to-treat” basis. 10. Results of statistical comparisons between groups were reported for at least one key outcome. 11. The study provides point measures and measures of variability for at least one key outcome. Scores correspond to 0 if the criterion is not met and 1 when it is met. Total scores equal to or greater than 7 are considered “high” quality, between 5 and 6 is considered “acceptable”, and 4 or lower is considered “poor”.

**Table 3:** Characteristics and main results of the studies.

Study	Population	Sample Size at the Beginning of the Study (N)	Study Design	Type of Intervention; Control Group	Delivery Times	Moments of the Study Evaluation	Result of Interest (Scale); Efficiency
Adler et al. (2008) [61]	U.S. peacekeepers	952	RCT of clusters	Single session of critical incident stress debriefing (CISD) 2. Single session of stress management class (SMC) 3.	Delivered in the last month of deployment at the processing facility	T1: Before deployment. T2: Last day of deployment. T3: 3 to 4 months. T4: 8 to 9 months after intervention.	PTSD (LCP): No significant effect for CISD compared with SMC or the no-intervention control. At high levels of exposure, there was a significant effect for CISD compared with SMC (*p* < 0.01), and for the no-intervention control compared with SMC from baseline to 3-month follow-up (*p* < 0.05). Depression (CES-D): No significant effect.
Tuckey and Scott (2014) [62]	Emergency workers (volunteer firefighters) through employee assistance program (EAP)	67	ECA	The approximately 90 min CISD sessions followed the seven-phase protocol of Mitchell [29]: (1) introduction, (2) facts, (3) thoughts, (4) reactions, (5) symptoms, (6) education, and (7) reengagement; screening (i.e., no treatment), stress, anagement education.	An invitation to participate in the study was issued in response to all requests for post-PAS interventions made to the PAD team coordinator during the sampling period: September 2007 to February 2009	T1: Invitation to participate in the study in response to all post-PTE intervention requests made to the EAP team coordinator. T2: After initial contact and consent, the brigade was randomly assigned to one of three intervention conditions: (1) CISD, (2) stress management education, and (3) screening. T3: Follow-up. T4: Analysis	There were no significant effects on post-traumatic stress or psychological distress. Overall, CISD may benefit broader functioning after exposure to work-related PTEs.
Deahl et al. (2000) [63]	Soldiers	106		(N = 54) received a formal PD of approximately 2 h, according to the Mitchell and Dyregrov method; the second group (N = 52) did not receive a formal PD of approximately 2 h, according to the Mitchell and Dyregrov method.	Immediately after return from Bosnia	T1: After their 6-month operational mission in Bosnia, the soldiers were randomly distributed into two groups. T2: Immediately after their return from Bosnia, the first group received a formal PD, while the second did not. T3: All soldiers completed a demographic questionnaire.	CAGE questionnaire scores decreased significantly in the group that received debriefing at the end of the follow-up period.
Macnab et al. (2003) [64]	Paramedics and emergency medical technicians (EMTs)	62	RCT of three levels of intervention in critical stress.	The mild intervention consisted of “listening” over the telephone and consulting a brochure describing post-traumatic stress symptoms. A moderate intervention consisted of immediate “listening”, consulting the brochure, and referral to a post-traumatic stress coordinator for a debriefing. A severe intervention was only relevant if more than one person involved in an event experienced CIS. A severe intervention consisted of de-escalation with others involved in the incident, and subsequent debriefing with a critical incident stress coordinator.	During the 6 months following the start of the study. Mild intervention: by telephone; moderate intervention: immediately by telephone; severe intervention: after the telephone call.	T1: Definition of CIS work and elaboration of intervention protocols. T2: Reception of telephone calls and random assignment to “mild”, “moderate”, or “severe” intervention. T3: Interventions. T4: Follow-up	Requests for post-traumatic stress intervention were infrequent. There was no correlation [64] between incident severity and scores on the Stanford Acute Stress Reaction, Impact of Events, or Life Impact Score Questionnaires, or between any of the scores. There was no consistent pattern in stress scores over time among the six subjects who completed all questionnaires.
Adler et al. (2009) [43]	Soldiers	2297	ECA	Single session of Battlemind debriefing 2. Single session of small-group Battlemind training 3. Single session of large-group Battlemind training 4. Single session of stress education (active management). Single session of stress education (active management) 5.	Several days after the deployment	T1: A few days after returning from deployment T2: 4 months	PTSD (PCL): No significant effects were identified. However, in subgroups with high exposure, Battlemind debriefing was found to have a significant impact (*p* < 0.05), as was Battlemind training in small and large groups compared with stress education (*p* < 0.001 and *p* < 0.01). Depression (PHQ-D): Battlemind training in large groups showed a significant impact compared to stress education (*p* < 0.05). In addition, debriefing also had a significant effect on those with high exposure (*p* < 0.05).
Wu et al. (2012) [49]	Chinese military rescuers	1267	RCT of clusters	Single session of psychological debriefing and training in team cohesion (512 PIM) 2. Single session of psychological debriefing (PD) 3.	Approximately 1 month after the traumatic incident	T1: 1 month after the earthquake; T2: 1 month; T3: 2 months; T4: 4 months	PTSD (SI-PTSD): Symptoms decreased over time in all groups. The 512 PIM group had significantly lower scores than the other two conditions at T3 and T4 (*p* < 0.01 for both time points). There were no significant differences between the debriefing and control groups. Anxiety and depression (Chinese translations of the HADS): Decreased over time in all groups. 512 PIM led to a greater reduction in anxiety (*p* < 0.01) and depression (*p* < 0.01) from T1 to T4 relative to the other conditions. There were no significant differences between the debriefing and control groups in anxiety or depression.

RCT: randomized controlled trial, CISD: critical incident stress debriefing, SMC: stress management class, PTSD: post-traumatic stress disorder, T1: data collection 1, T2: data collection 2, T3: data collection 3, T4: data collection 4, EAP: employee assistance program, PTE: potentially traumatic event, PD: psychological debriefing, CIS: critical incident stress, PIM: psychological intervention model.

## Data Availability

Data are contained within the article.

## References

[1] Testa V., Heber A., Groll D., Ritchie K., Tam-Seto L., Mulligan A., Sullo E., Schick A., Bose E., Jabbari Y. (2023). Glossary of terms: A shared understanding of the common terms used to describe psychological trauma, version 3.0. Health Promot. Chronic Dis. Prev. Can..

[2] Oliphant R. (2016). Healthy Minds, Safe Communities, Supporting Our Public Safety Officers through a National Strateegy for Operational Stress Injuries: Report of the Standing Committee on Public Safety and National Security.

[3] Courtney J.A., Francis A.J.P., Paxton S.J. (2013). Caring for the country: Fatigue, sleep and mental health in Australian rural paramedic shiftworkers. J. Community Health.

[4] Cramm H., Richmond R., Jamshidi L., Edgelow M., Groll D., Ricciardelli R., MacDermid J.C., Keiley M., Carleton R.N. (2021). Mental Health of Canadian Firefighters: The Impact of Sleep. Int. J. Environ. Res. Public Health.

[5] Sun X., Wang Z., Liu H., Ren M., Feng D. (2022). Physical and mental health problems of Chinese front-line healthcare workers before, during and after the COVID-19 rescue mission: A qualitative study. BMJ Open.

[6] Bo Y., Liu H., Zhang M., He J., Miao C., Sha R., Yu H. (2023). Knowing the Psychological Risks of Anti-epidemic Rescue Teams for COVID-19 by Simplified Risk Probability Scale. Preprint.

[7] Carleton R.N., Afifi T.O., Taillieu T., Turner S., Krakauer R., Anderson G.S., MacPhee R.S., Ricciardelli R., Cramm H.A., Groll D. (2019). Exposures to potentially traumatic events among public safety personnel in Canada. Can. J. Behav. Sci..

[8] Smid G.E., Lind J., Bonde J.P. (2022). Neurobiological mechanisms underlying delayed expression of posttraumatic stress disorder: A scoping review. World J. Psychiatry.

[9] Galatzer-Levy I.R., Madan A., Neylan T.C., Henn-Haase C., Marmar C.R. (2011). Peritraumatic and trait dissociation differentiate police officers with resilient versus symptomatic trajectories of posttraumatic stress symptoms. J. Trauma Stress.

[10] Carleton R.N., Afifi T.O., Turner S., Taillieu T., Duranceau S., LeBouthillier D.M., Sareen J., Ricciardelli R., Macphee R.S., Groll D. (2018). Mental Disorder Symptoms among Public Safety Personnel in Canada. Can. J. Psychiatry.

[11] Williamson J.B., Jaffee M.S., Jorge R.E. (2021). Posttraumatic Stress Disorder and Anxiety-Related Conditions. Contin. Lifelong Learn. Neurol..

[12] Pary R., Micchelli A.N., Lippmann S. (2021). How We Treat Posttraumatic Stress Disorder. Prim Care Companion CNS Disord..

[13] Ayuso Mateos J.L., Vieta Pascual E., Arango López C., Bagney Lifante A., American Psychiatric Association (2014). DSM-5: Manual Diagnóstico y Estadístico de Los Trastornos Mentales, 5a.

[14] Gabriel R., Ferrando L., Cortón E.S., Mingote C., García-Camba E., Liria A.F., Galea S. (2007). Psychopathological consequences after a terrorist attack: An epidemiological study among victims, the general population, and police officers. Eur. Psychiatry.

[15] Boffa J.W., Stanley I.H., Smith L.J.B., Mathes B.M.B., Tran J.K., Buser S.J., Schmidt N.B., Vujanovic A.A. (2018). PTSD Symptoms and Suicide Risk in Male Firefighters: The Mediating Role of Anxiety Sensitivity. J. Nerv. Ment. Dis..

[16] Hoell A., Kourmpeli E., Dressing H. (2023). Work-related posttraumatic stress disorder in paramedics in comparison to data from the general population of working age. A systematic review and meta-analysis. Front. Public Health.

[17] Skeffington P.M., Rees C.S., Mazzucchelli T. (2017). Trauma exposure and post-traumatic stress disorder within fire and emergency services in Western Australia. Aust. J. Psychol..

[18] Tomaka J., Magoc D., Morales-Monks S.M., Reyes A.C. (2017). Posttraumatic Stress Symptoms and Alcohol-Related Outcomes Among Municipal Firefighters. J. Trauma Stress.

[19] Statista Number of Post-Traumatic Stress Disorder (PTSD) Cases Registered in Spain from 2011 to 2022; Statista Research Department. https://www.statista.com/statistics/1094684/number-of-cases-of-posttraumatic-stress-spain/.

[20] Katsavouni F., Bebetsos E., Malliou P., Beneka A. (2016). The relationship between burnout, PTSD symptoms and injuries in firefighters. Occup. Med..

[21] Almutairi M.N., Azza A.A. (2020). Burnout and coping methods among emergency medical services professionals. J. Multidiscip. Healthc..

[22] WHO (2019). Burn-out an “occupational phenomenon”: International Classification of Diseases. WHO Departmental News.

[23] Stevelink S.A.M., Pernet D., Dregan A., Davis K., Walker-Bone K., Fear N.T., Hotopf M. (2020). The mental health of emergency services personnel in the UK Biobank: A comparison with the working population. Eur. J. Psychotraumatol..

[24] Nutt J.D., Stein B.M., Zohar J., Informa Health Care (2009). Post Traumatic Stress Disorder.

[25] Gesteira C., García-Vera M.P., Sanz J., Gesteira C., García-Vera M.P., Sanz J. (2018). Porque el Tiempo no lo Cura Todo: Eficacia de la Terapia Cognitivo-conductual Centrada en el Trauma para el Estrés postraumático a muy Largo Plazo en Víctimas de Terrorismo. Clin. Salud.

[26] Kar N. (2011). Cognitive behavioral therapy for the treatment of post-traumatic stress disorder: A review. Neuropsychiatr. Dis. Treat..

[27] Coimbra M.A.R., Ikegami É.M., Souza L.A., Haas V.J., Barbosa M.H., Ferreira L.A. (2024). Eficacia de un programa en el aumento de las estrategias de coping en bomberos: Ensayo clínico aleatorizado. Rev. Lat. Am. Enferm..

[28] Mitchell J.T., Everly G.S. (2001). Critical Incident Stress Management: An Operations Manual for CISD, Defusing and Other Group Crisis Intervention Services.

[29] Mitchell J.T., Everly G.S. (1993). Critical Incident Stress Debriefing. https://corpslakes.erdc.dren.mil/employees/cism/pdfs/Debriefing.pdf.

[30] World Health Organization (2013). Guidelines for the Management of Conditions Specifically Related to Stress. https://www.who.int/publications/i/item/9789241505406.

[31] Basiaga-Pasternak J., Pomykała S., Cichosz A. (2015). Stress in volunteer mountain rescue teams. Stud. Sport Humanit..

[32] DiMaggio C., Madrid P.A., Loo G.T., Galea S. (2008). The Mental Health Consequences of Terrorism: Implications for Emergency Medicine Practitioners. J. Emerg. Med..

[33] Devilly G.J., Cotton P. (2003). Psychological debriefing and the workplace: Defining a concept, controversies and guidelines for intervention. Aust. Psychol..

[34] Malcolm A.S., Seaton J., Perera A., Sheehan D.C., Van Hasselt V.B. (2005). Critical incident stress debriefing and law enforcement: An evaluative review. Brief Treat. Crisis Interv..

[35] Hawker D.M., Durkin J., Hawker D.S.J. (2011). To debrief or not to debrief our heroes: That is the question. Clin. Psychol. Psychother..

[36] Anderson E., Sandars J., Kinnair D. (2019). The nature and benefits of team-based reflection on a patient death by healthcare professionals: A scoping review. J. Interprofessional Care.

[37] Sandoval J.B., Hooshmand M., Sarik D.A. (2023). Beating Burnout with Project D.E.A.R.: Debriefing Event for Analysis and Recovery. Nurse Lead.

[38] Castro R., Campero L., Hernández B. (1997). La investigación sobre apoyo social en salud: Situación actual y nuevos desafíos. Rev. Saude Publica.

[39] Durá E., Garcés J. (1991). La teoría del apoyo social y sus implicaciones para el ajuste psicosocial de los enfermos oncológicos. Rev. Psicol. Soc..

[40] Aulagnier M., Verger P., Rouillon F. (2004). Efficacité du « débriefing psychologique » dans la prévention des troubles psychologiques post-traumatiques: Efficiency of psychological debriefing in preventing post-traumatic stress disorders. Rev. Epidemiol. Sante Publique.

[41] Sandhu G., Colon J., Barlow D., Ferris D. (2016). Daily Informal Multidisciplinary Intensive Care Unit Operational Debriefing Provides Effective Support for Intensive Care Unit Nurses. Dimens. Crit. Care Nurs..

[42] Tamrakar T., Murphy J., Elklit A. (2019). Was Psychological Debriefing Dismissed Too Quickly?. Crisis Stress Hum. Resil. Int. J..

[43] Adler A.B., Bliese P.D., McGurk D., Hoge C.W., Castro C.A. (2009). Battlemind debriefing and battlemind training as early interventions with soldiers returning from Iraq: Randomization by platoon. J. Consult. Clin. Psychol..

[44] Shipherd J.C., Salters-Pedneault K., Fordiani J. (2016). Evaluating postdeployment training for coping with intrusive cognition: A comparison of training approaches. J. Consult. Clin. Psychol..

[45] Mulligan K., Fear N.T., Jones N., Wessely S., Greenberg N. (2011). Psycho-educational interventions designed to prevent deployment-related psychological ill-health in Armed Forces personnel: A review. Psychol. Med..

[46] Schell T.L., Farris C., Miles J.N.V., Sloan J., Scharf D.M. (2017). The Air Force Deployment Transition Center: Assessment of Program Structure, Process, and Outcomes. Rand Health Q..

[47] Acosta J., Becker A., Cerully J., Fisher M., Martin L., Vardavas R., Slaughter M., Schell T. (2014). Mental Health Stigma in the Military.

[48] Nash W.P., Krantz L., Stein N., Westphal R.J., Litz B. (2011). Comprehensive soldier fitness, battlemind, and the stress continuum model: Military organizational approaches to prevention. Caring for Veterans with Deployment-Related Stress Disorders.

[49] Wu S., Zhu X., Zhang Y., Liang J., Liu X., Yang Y., Yang H., Miao D. (2012). A new psychological intervention: “512 Psychological Intervention Model” used for military rescuers in Wenchuan Earthquake in China. Soc. Psychiatry Psychiatr. Epidemiol..

[50] Hong C., Efferth T. (2016). Systematic Review on Post-Traumatic Stress Disorder Among Survivors of the Wenchuan Earthquake. Trauma Violence Abuse.

[51] Winders W.T., Bustamante N.D., Garbern S.C., Bills C., Coker A., Trehan I., Osei-Ampofo M., Levine A.C. (2021). Establishing the Effectiveness of Interventions Provided to First Responders to Prevent and/or Treat Mental Health Effects of Response to a Disaster: A Systematic Review. Disaster. Med. Public Health Prep..

[52] Arancibia M., Leyton F., Morán J., Muga A., Ríos U., Sepúlveda E., Vallejo-Correa V. (2022). Psychological debriefing in acute traumatic events: Evidence synthesis. Medwave.

[53] Vignaud P., Lavallé L., Brunelin J., Prieto N. (2022). Are psychological debriefing groups after a potential traumatic event suitable to prevent the symptoms of PTSD?. Psychiatry Res..

[54] Rose S.C., Bisson J., Churchill R., Wessely S. (2000). Brief psychological interventions (“debriefing”) for trauma-related symptoms and the prevention of post traumatic stress disorder. Cochrane Database Syst. Rev..

[55] Wesemann U., Mahnke M., Polk S., Bühler A., Willmund G. (2020). Impact of Crisis Intervention on the Mental Health Status of Emergency Responders Following the Berlin Terrorist Attack in 2016. Disaster. Med. Public Health Prep..

[56] Yepes-Nuñez J.J., Urrútia G., Romero-García M., Alonso-Fernández S. (2021). Declaración PRISMA 2020: Una guía actualizada para la publicación de revisiones sistemáticas. Rev. Esp. Cardiol..

[57] Higgins JP T., Thomas J., Chandler J., Cumpston M., Li T., Page M.J., Welch V.A. (2023). Manual Cochrane Para Revisiones Sistemáticas de Intervenciones, Versión 6.4 (Actualizado En Agosto de 2023).

[58] Santos C.M.D.C., Pimenta C.A.D.M., Nobre M.R.C. (2007). Estrategia PICO para la construcción de la pregunta de investigación y la búsqueda de evidencias. Rev. Lat. Am. Enferm..

[59] Verhagen A.P., de Vet H.C., de Bie R.A., Kessels A.G., Boers M., Bouter L.M., Knipschild P.G. (1998). The Delphi list: A criteria list for quality assessment of randomized clinical trials for conducting systematic reviews developed by Delphi consensus. J. Clin. Epidemiol..

[60] Page M.J., McKenzie J.E., Bossuyt P.M., Boutron I., Hoffmann T.C., Mulrow C.D., Shamserm L., Tetzlaff J.M., Akl E.A., Brennan S.E. (2021). The PRISMA 2020 statement: An updated guideline for reporting systematic reviews. BMJ.

[61] Adler A.B., Litz B.T., Castro C.A., Suvak M., Thomas J.L., Burrell L., McGurk D., Wright K.M., Bliese P.D. (2008). A group randomized trial of critical incident stress debriefing provided to U.S. peacekeepers. J. Trauma Stress.

[62] Tuckey M.R., Scott J.E. (2014). Group critical incident stress debriefing with emergency services personnel: A randomized controlled trial. Anxiety Stress Coping.

[63] Deahl M., Srinivasan M., Jones N., Thomas J., Neblett C., Jolly A. (2000). Preventing psychological trauma in soldiers: The role of operational stress training and psychological debriefing. Br. J. Med. Psychol..

[64] Macnab A., Sun C., Lowe J. (2003). Randomized, controlled trial of three levels of critical incident stress intervention. Prehospital Disaster Med..

[65] Lawrence-Wood E., Van Hooff M., McFarlane A. (2021). Anger in occupations characterized by repeated threat and stress exposure: The longitudinal view in the military context. Anger at Work: Prevention, Intervention, and Treatment in High-Risk Occupations.

[66] Van Mol M.M.C., Kompanje E.J.O., Benoit D.D., Bakker J., Nijkamp M.D., Seedat S. (2015). The Prevalence of Compassion Fatigue and Burnout among Healthcare Professionals in Intensive Care Units: A Systematic Review. PLoS ONE.

[67] Bisson J.I., Jenkins P.L., Alexander J., Bannister C. (1997). Randomised controlled trial of psychological debriefing for victims of acute burn trauma. Br. J. Psychiatry.

[68] Deng J., Kou X., Ma H., Niu A., Luo Y. (2024). Qualitative study on the core competencies of nursing personnel in emergency medical rescue teams at comprehensive hospitals in Chongqing, China. BMJ Open.

